# Oscillometric measure of blood pressure detects association between orthostatic hypotension and depression in population based study of older adults

**DOI:** 10.1186/1471-244X-13-266

**Published:** 2013-10-18

**Authors:** Claire O Regan, Patricia M Kearney, Hilary Cronin, George M Savva, Brian A Lawlor, Roseanne Kenny

**Affiliations:** 1 The Irish Longitudinal Study on Ageing (TILDA), Department of Medical Gerontology, Trinity College, Dublin, Ireland; 2 Department of Epidemiology and Public Health, University College Cork, Cork, Ireland; 3 Department of Psychiatry, Trinity College, Dublin, Ireland

## Abstract

**Background:**

White matter hyperintensities may contribute to depression by disrupting neural connections among brain regions that regulate mood. Orthostatic hypotension (OH) may be a risk factor for white matter hyperintensities and accumulating evidence, although limited suggests it may play a role in the development of late-life depression. The aim of this study was to examine the relationship between an oscillometric measure of orthostatic hypotension and depression in population based sample of older adults.

**Methods:**

We analysed data on adults aged 60 and over from the first wave of The Irish Longitudinal Study on Ageing (TILDA). Depression was assessed using the Center for Epidemiologic Studies – Depression (CES–D) scale and OH was assessed by a sit-to-stand orthostatic stress test; two seated blood pressure measurements were followed by a single standing blood pressure measurement. Participants self reported whether they felt dizzy, light-headed or unsteady on standing.

**Results:**

Participants with symptomatic OH (SOH, n=20) had the highest mean CES-D score (mean 8.6, SE 1.6) when compared to participants with asymptomatic OH (AOH) (mean 5.6, SE .48) and participants with no OH (mean 5.2, SE .14) and this difference was significant for both comparisons (p<0.001). Linear regression analysis adjusted for socio-demographic and clinical characteristics showed that SOH was associated with higher CES-D scores (unstandardised B coefficient = 2.24; 95% CI .301 - 4.79; p =0.05) compared to participants without OH. AOH was not associated with higher CES-D scores (unstandardised B coefficient =.162; 95% CI -.681, 1.00; p= 0.70).

**Conclusions:**

Symptomatic orthostatic hypotension is associated with depression in older adults and needs to be considered in studies examining the relationship between vascular disease and depression in older adults.

## Background

Depression is common in late life [1] and associated with diverse aetiological factors that are poorly understood. Evidence of an association between vascular disease and late-life depression (LLD) led to the 'vascular depression hypothesis’ which proposed that structural damage to the frontostriatal tracts resulting from ischemic cerebrovascular disease creates a vulnerability to depression in late life [2,3]. This hypothesis stimulated much research and laid the foundation for examining the mechanisms by which vascular disease influences the development of depression in older adults. As a result, a mechanistic model for vascular depression has recently been proposed that identifies hypoperfusion as a potential mechanistic path to vascular depression [4].

Blood flow to the brain is influenced by systemic hemodynamics and cerebrovascular autoregulation, whereby cerebral arteries contract or dilate in response to arterial blood pressure (BP) changes. These processes interact to maintain stable perfusion but are impaired in the context of vascular disease leading to perfusion deficits and potentially the development of white matter hyperintensities (WMHs) [4]. White matter is sensitive to transient ischemia because it is supplied by terminal arterioles with limited collateral flow and so is more susceptible to minor changes in flow due to impaired autoregulation and consequent infarction [5]. WMHs are believed to contribute to the pathogenesis of LLD since MRI studies have repeatedly found higher densities in the frontal lobes [6-8] and basal ganglia [8] of older people with major depression (MD) and these regions of the brain are known to regulate mood and cognition. Although the pathology of WMHs varies, autopsy studies have shown that they are associated with ischemic damage and tissue hypoxia in patients with late-life major depression [9]. In animal models white matter ischemia results from drops in blood pressure [10] and a recent study [11] has shown a relationship between the degree of orthostatic drop in BP and WMH volume in LLD.

Hemodynamic homeostasis becomes less effective with aging and is associated with a decreased ability to regulate BP [12]. Orthostatic hypotension (OH) is an excessive fall in BP after standing which may result from inadequate intravascular volume, autonomic nervous system dysfunction, decreased venous return or an inability to increase cardiac output in response to postural changes [13]. After essential hypertension, OH is the most common disorder of BP regulation and in adults aged 65 years and older the prevalence ranges between 5 and 30%; depending on the population studied and definition used [14]. Prospective studies have shown that OH is predictive of ischemic stroke [15] and all-cause mortality in older adults [16].

In 1996, a consensus committee defined classical OH as a drop of at least 20 mmHg in systolic blood pressure (SBP) or 10 mmHg in diastolic blood pressure (DBP) within the first 3 minutes of standing [17]. This may reduce perfusion pressure of organs, especially above heart level, such as the brain. OH can be asymptomatic or manifest as symptoms that range from dizziness and light-headedness to weakness and loss of consciousness. These symptoms arise from periods of hypoperfusion that have the potential to induce ischemic damage and WMHs [14,18]. For this reason, it has been suggested that OH may play an important role in the development of depression and accumulating evidence, although limited shows that OH may be more frequent in late-life depression [19,20]. Previous studies confirming an association between OH and depression were conducted on small clinical cohorts of patients with a formal diagnosis of MD and using hospital based digital photoplethysmographic measures of phasic BP. It is not known whether the association would persist in the general population; where lower prevalence and severity rates for these conditions are observed, and using traditional oscillometric measures of BP. Consequently, the aim of this study is to investigate if OH is associated with depression in a nationally representative sample of older adults utilising a standard test of OH and a self-report measure of depression.

## Methods

### Study design

We analysed data from the first wave of The Irish Longitudinal Study on Ageing (TILDA) collected between October 2009 and February 2011. Full details of the sampling procedure and response have been described elsewhere [21]. In short TILDA is a study of community dwelling adults (nationally representative sample) aged 50 and over. Participants completed a computer-assisted personal interview (CAPI) in their own homes which included detailed questions on health, social and economic circumstances. Each participant was then invited to a health centre for a comprehensive health assessment. Participants who were unable or unwilling to attend a health centre were offered a modified assessment in their own home. People with known or suspected dementias were ineligible at baseline for participation. All assessments were carried out by trained nurses. The study was approved by the Faculty of Health Sciences Research Ethics Committee at Trinity College Dublin and all participants gave informed written consent. All experimental procedures adhered to the Declaration of Helsinki. The measures specific to the current analysis are described below.

### Neuropsychiatric assessment

The primary outcome measure for this analysis was the mean score on the 20-item Centre for Epidemiologic Studies Depression scale (CES-D). The CES-D generates a total score with a range between 0 and 60 with higher scores indicating greater depressive symptoms. A cut-off of 16 has been shown to have a sensitivity of 100% and a specificity of 88% for MD in older adults [22,23]. LLD is commonly defined as depression occurring in adults aged 60 or 65 years and over therefore only participant’s ≥ 60 years were included in the analysis. Participants taking antidepressants were excluded from this study.

Anxiety was assessed using the Hospital Anxiety Depression Scale – Anxiety subscale. A cut-off of ≥11 was used to classify participants with anxiety [24]. The MMSE (Mini Mental State Examination) was used to assess cognition [25].

### Blood pressure measurement

Participants underwent a sit-to-stand orthostatic stress test (STST).

#### Seated blood pressure

Two seated SBP and DBP measurements were obtained 1 minute apart using an OMRON™ digital automatic blood pressure monitor (Model M10-IT). Participants had been seated for at least 30 minutes and were a minimum of 1 hour pre or post lunch when the measurement was obtained. The means of the two sitting SBP and DBP readings were used in the analysis.

#### Standing blood pressure

Immediately after the second seated BP measurement, the participant was asked to stand and a single SBP and DBP measurement was obtained, using the same monitor with the cuff at heart level. Immediately after the standing BP measurement was complete, participants were asked to report whether they had felt dizzy, light-headed or unsteady on standing (yes or no to any of the symptoms).

##### Asymptomatic orthostatic hypotension

Participants with a reduction of SBP of at least 20 mmHg or DBP of 10 mmHg without symptoms of dizziness/light-headedness after standing were classified as having asymptomatic OH (AOH).

##### Symptomatic orthostatic hypotension

Participants with a reduction of SBP of at least 20 mmHg or DBP of 10 mmHg accompanied by symptoms of dizziness/light-headedness after standing were classified as having symptomatic OH (SOH).

### Other measures

Other measures recorded during the home interview (CAPI) included age, gender, highest level of educational attainment (primary, secondary or tertiary), current smoking status, history of cardiovascular disease and medication use. Medication use was determined by recording medication names from the medicine bottles in the participant’s home during the CAPI interview. Medications were classified using the World Health Organization Anatomical Therapeutic Chemical (ATC) system [26]. A dichotomous variable for antihyperintensive medication was computed (1=yes, 0=no) with the following ATC codes: C02; diuretic drugs, C03; peripheral vasodilator drugs, C04; vasoprotective drugs, C05; β-blocking agents, C07; and calcium-channel blockers C08. Psychotropic medications were classified by ATC code N05. In addition to the beat-to-beat BP measurements SBP and DBP were recorded during seated rest using a digital automatic BP monitor (OMRON Model M10-IT). For descriptive purposes, participants were classified as hypertensive if the mean of their two seated SBP measurements was ≥140 mmHg and/or if the mean of their two seated DBP measurements was ≥90 mmHg or if they were currently taking antihypertensive medications [27]. Objective measures of weight (1 measure using SECA electronic floor scales) and height (1 measure using SECA 240 wall mounted measuring rod) were used to calculate body mass index (BMI). Total cholesterol was determined from a venous blood sample.

### Statistical analysis

Only participants who completed a health assessment were eligible for inclusion. Statistical analysis was performed using SPSS version 20. Distribution of continuous variables was assessed using Q-Q plots and histograms. Normally distributed variables were described as means and standard errors (SE), and were compared using independent t-tests and categorical variables were compared using Chi-squared tests. AOH and SOH and were analysed separately a priori.

Multivariate linear regression analysis was used to assess the relationship between OH and depression with adjustment for potential confounders including age, sex, education level, MMSE, history of cardiovascular disease (angina, stroke, TIA, myocardial infarction), total cholesterol, smoking (0=non/previous smoker, 1=current smoker), BMI, SBP and DBP values, antihypertensive and psychotropic medications as covariates. Unstandardised regression coefficients, 95% confidence intervals and significance levels are presented here. Differences with p<0.05 (two-tailed) were considered statistically significant. Sensitivity analysis was conducted to explore the impact of including participants on antidepressant medications. Data was weighted with respect to age, sex and education to the Quarterly National Household Survey (2010) and further weighted to account for those who did not attend for a health assessment (see TILDA wave 1 report for further information on the calculation of weights, [http://www.tilda.ie]).

## Results

### Characteristics of the sample

**Figure 1:**
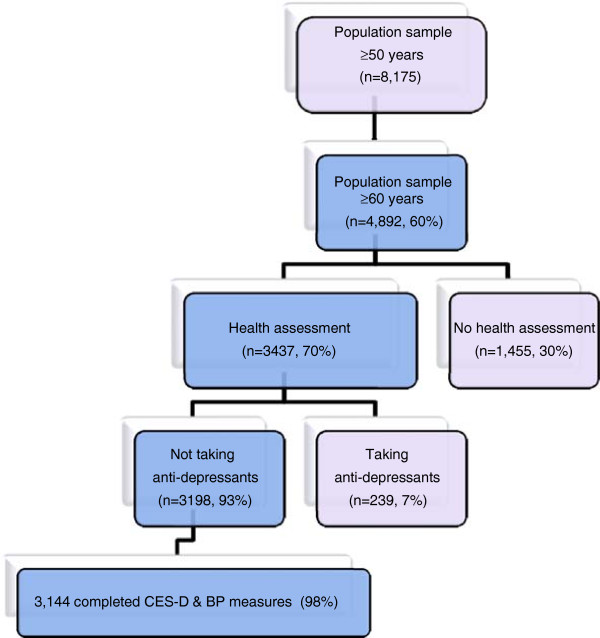
TILDA sample used in current analysis.

At baseline 8,175 participants were recruited to the TILDA study and the household response rate was 62%. 4,892 individuals were aged 60 and over and 3,437 of these completed a health assessment. The study population used in this analysis is depicted in Figure 1. In total 3,144 participants were eligible for the current analyses. Of these, 52% were female with a mean age of 70.0, (SE .01, range 60–98).

Table 1 shows demographic and clinical characteristics of participants by orthostatic blood pressure group. 8% (n=240) of participants in our study had OH and 9% (n= 20) of these reported symptoms of dizziness, light-headedness or unsteadiness on standing. Symptoms were only reported by people who were classified with OH. Participants with OH were more likely to be female (p=0.01), had significantly more hypertension (p<0.001), lower BMI (p=0.01) and a lower MMSE score (p=0.04).

**Table 1:** Demographic and clinical characteristics overall and by OH group

	**Overall**		**No OH**		**OH**		**P-value**^**h**^	
	**N=3144**		**N=2904**		**N=240**			
**Age** mean (SE)	69.3	(.18)	69.1	(.17)	71.6	(.59)	t= -4.29	<0.001
**Female sex** n (%)	1650	52.0	1506	51.2	144	61.8	X^2=^ 5.89	0.01
**Education**: n (%)								
Primary	1075	44.0	988	43.6	87	49.2	X^2^= .489	0.48
Secondary	1141	38.3	1062	38.6	79	33.5	X^2^= 1.29	0.26
Tertiary	926	17.7	852	17.7	74	17.4	X^2^= .232	0.65
**Current smoker** n (%)	393	13.2	360	13.1	33	14.4	X^2^= .371	0.54
**Hypertension**^**a**^ n (%)	1830	59.5	1667	58.7	163	69.4	X^2^= 10.5	0.002
**Antihypertensive medications** n (%)	1466	47.3	1324	46.7	122	54.3	X^2^= 3.14	0.08
**Psychotropic medication** n (%)	193	6.1	181	6.2	12	5.0	X^2^=585	0.44
**History cardiovascular disease**^**b**^ n (%)	389	13.1	368	13.4	21	9.6	X^2^= 3.14	0.08
**Diabetes** n (%)	270	8.8	249	8.7	21	9.5	X^2=^.009	0.90
**Anxiety**^**c**^ n (%)	150	5.7	138	5.6	12	5.9	X^2^= .036	0.87
**Orthostatic symptoms**^**d**^ n (%)	20	8.5	0	0	20	8.5	X^2^=243	<0.001
**High cholesterol**^**e**^ mean (SE)	4.95	(.02)	4.95	(.07)	4.99	(.07)	t= -.627	0.53
**BMI**^**f**^ mean (SE)	27.7	(.05)	28.7	(.09)	27.8	(.19)	t=2.35	0.01
**MMSE**^**g**^ mean (SE)	27.3	(.19)	28.0	(.19)	27.2	(.88)	t=2.12	0.04

^**a**^mean of 2 seated systolic blood pressures is ≥140 mmHg and/or mean of 2 seated diastolic blood pressures is ≥90 mmHg or currently taking antihypertensive medications.

^**b**^history of angina, stroke or myocardial infarction.

^**c**^Hospital Anxiety and Depression Scale ≥ 11 (range 0–21).

^**d**^symptoms of dizziness, lightheaded or unsteadiness on standing.

^**e**^measure obtained from venous sample.

^**f**^Body Mass Index.

^**g**^Mini Mental State Examination (range 0–30).

^**h**^comparison of no OH and OH group based on t-test for continuous variables and X^2^ for categorical variables.

### OH and depression

**Figure 2:**
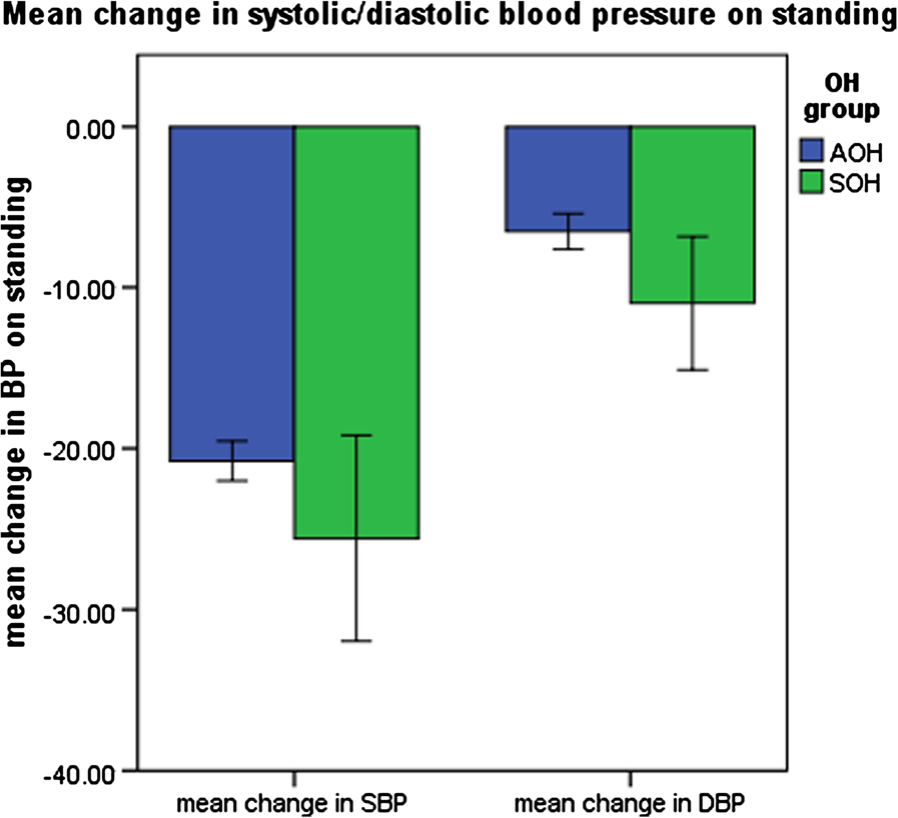
Mean change in systolic/diastolic blood pressure on standing.

**Figure 3:**
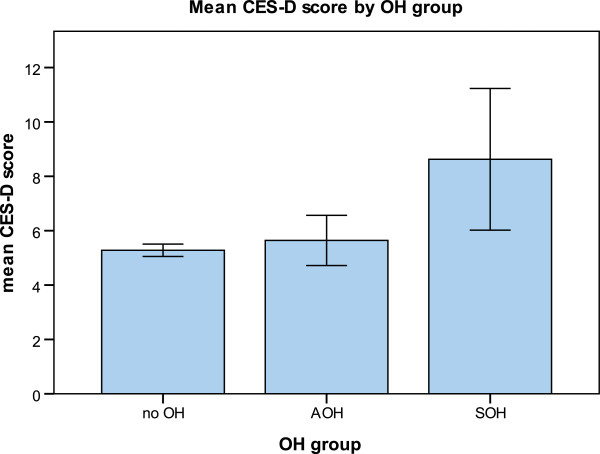
Mean CES-D score by OH group.

Table 2 shows BP measurements in participants with AOH and symptomatic SOH. Participants with SOH had greater drops in SBP and DBP compared to participants with AOH (see Figure 2). Figure 3 shows mean CES-D score by OH group. Participants with SOH had the highest mean CES-D score (mean 8.6, SE 1.6) when compared to participants with AOH (mean 5.6, SE .48) and participants without OH (mean 5.2, SE .14). In multivariate linear regression analysis adjusted for socio-demographic and clinical characteristics, SOH was associated with higher levels of depressive symptoms (see Table 3). Mean CES-D score was on average 3 points higher in participants with SOH compared to participants without OH. AOH was not associated with significantly higher CES-D scores. Sensitivity analysis including participants on antidepressants attenuated but did not eliminate the significant association between SOH and depression (unstandardised B coefficient = 1.4, 95% CI .11-3.9). The sensitivity model included all covariates from model 3 and additionally controlled for antidepressant use.

**Table 2:** Blood pressure parameters by OH group

	**Asymptomatic OH**		**Symptomatic OH**		
	**N=220**		**N= 20**		
	**Mean**	**SE**	**Mean**	**SE**	**t-test**
**Seated systolic BP**^**a**^	150.8	(1.4)	146.8	(4.1)	t=.996 p=0.320
**Seated diastolic BP**^**a**^	86.8	(.80)	87.4	(2.8)	t=-.073 p=.942
**Standing systolic BP**	129.5	(1.5)	121.1	(4.0)	t=-1.87 p=.062
**Standing diastolic BP**	79.50	(.85)	77.15	(2.1)	t=-1.53 p=.127
**Mean change in SBP**^**b**^	-21.30	(.78)	-25.65	(.23)	t=-1.75 p=.080
**Mean change in DBP**^**c**^	-7.30	(.63)	-10.25	(.14)	t=-1.89 p=.050

^a^average of 2 readings.

^b^mean of 2 seated SBP readings subtracted from 1 standing SBP reading.

^c^mean of 2 seated DBP readings subtracted from 1 standing DBP reading.

**Table 3:** Linear regression analysis examining the effect of OH on CES-D score

	**Model 1**^**b**^			**Model 2**^**c**^			**Model 3**^**d**^		
	**B coeff**^**e**^**95% CI**		**p**	**B coeff 95% CI**		**p**	**B coeff 95% CI**		**p**
**Asymptomatic OH**^a^	0.333	-.499-1.16	0.40	0.199	-.624-1.02	0.635	0.162	-.681-1.00	0.70
**Symptomatic OH**^a^	2.83	.173-5.48	0.03	2.71	.091-5.33	0.042	2.24	.301- 4.79	0.05

^a^comparsion group is participants without OH (coefficient for AOH and SOH modeled concurrently).

^b^univariate analysis.

^c^adjusted for age, sex, education.

^d^adjusted for age, sex, education, MMSE, History CVD, blood cholesterol, smoking, BMI, heart.

and psychotropic medications, seated systolic BP and seated diastolic BP.

^e^unstandardised B coefficient.

## Discussion

In this study, we found evidence of a relationship between orthostatic hypotension and depression in participants with OH who reported symptoms of hypoperfusion on standing. Emerging evidence suggests that hypoperfusion may be an important factor in the development of late-life vascular depression [4]. OH can lead to cerebral auto-dysregulation [28] which can impair cerebral blood flow (CBF) [29]. Perfusion deficits do not need to cause ischemia in order to influence brain function. Mild CBF reduction impairs protein synthesis [30] that is crucial for cognitive and affective processing while greater CBF reduction in the context of autoregulatory deficits may cause ischemic injury and WMHs [4]. Previous results linking OH and depression have been observed among clinical samples [11,19,20] where cases of OH and depression are respectively more prevalent and severe. Our findings suggest that in a representative sample of community dwelling older adults, the relationship is limited to those with more severe OH; since the association is dependent on the presence of symptoms of hypoperfusion.

9% of participants with OH reported symptoms of dizziness/light-headedness on standing. Cerebral autoregulation (CA) explains why OH does not produce symptoms in all adults. Under normal physiologic conditions, changes in mean arterial BP between 60 and 160 mmHg produce little or no change in CBF. Beyond these limits, a sudden decrease in CBF occurs at the lower limit of autoregulation and likely manifests as orthostatic symptoms [29]. In patients with OH, severe autoregulatory failure is believed to occur in approximately one in four patients and cerebral hypoperfusion occurs in response to relatively small changes in BP [29]. Participants in our study with symptomatic OH may have impaired CA and more extreme changes in CBF resulting in orthostatic symptoms. In contrast, those with asymptomatic OH may have better preserved autoregulation centrally and therefore no symptoms of hypoperfusion from reduced CBF, despite clinically significant drops in BP during standing. While a causal relationship between WMH and cerebral hypoperfusion induced by OH has yet to be established, both animal models and human studies suggest that this exists [31,32] and future waves of TILDA will investigate if OH at baseline predicts depression at follow up.

The prevalence of hypertension was higher in participants with OH which could have confounded results since hypertension is a known risk factor for OH [33] and WMHs [34]. However, hypertension was not associated with increased CES-D score in univariate analysis and the addition of systolic and diastolic BP levels to the model only slightly attenuated the association between SOH and depression. The use of antihypertensive medication was also somewhat higher in the OH group; however, the relationship between SOH and depression also persisted even after adjusting for these medications in the final model. In healthy community-dwelling adults such as our participants these drugs are associated with only low rates of orthostatic hypotension [11,35] making them unlikely to have influenced our findings.

Participants currently taking antidepressants were excluded from our analysis. There is no simple way to 'adjust’ for anti-depressant use in our baseline data since the model we are trying to estimate is essentially cyclical (i.e. our hypothesis is that OH causes depression, but we know depression results in anti-depressant use and anti-depressant use can cause OH).

7% of our target population were currently taking antidepressants (see Figure 1). Sensitivity analysis including participants on antidepressants (and controlling for antidepressants in the model) attenuated but did not eliminate the significant association between SOH and depression and overlapping confidences intervals between estimates derived from the sample with antidepressant users, and without them, suggests no significant difference between estimates .

The STST is commonly used in everyday clinical practice, however it is believed to have inferior sensitivity for detecting OH compared to other orthostatic stress tests (Active Stand Test or the Head-Up Tilt-Table Test) [36]. Automated BP machines can take 25–40 seconds to record a measurement. We therefore expect that our findings underestimate the prevalence of OH (compared with prevalence rates estimated from phasic BP measurement) and potentially any associations uncovered; given that in some participants BP may have recovered before the recording was completed. Interestingly, orthostatic symptoms were only reported by participants with OH which would suggest that the STST has good sensitivity for detecting those with severe OH. Previous studies linking OH and depression have utilised continuous, beat-to-beat BP monitoring to measure OH in patients with MD at centre based assessments. Home respondents are known to differ markedly from the centre based attendees and in a TILDA pilot study we showed that participants who selected a home assessment had poorer physical health with higher levels depressive symptomology [37]. The STST is easily administered in an individual’s home therefore it maximised the participation of older participants in our study and was effective for identifying the association between symptomatic OH and depression.

The main strengths of this study are the large population representative sample and comprehensive health assessment. Limitations are that the data was analysed cross-sectionally so we cannot make any direct inferences about temporality. Although many relevant risk factors were assessed and statistically controlled for in the analysis, the possibility of residual confounding exists. In particular psychotrophic medications are captured by a single variable which could pool agents that cause OH with those that do not. Depression was not formally diagnosed in our study although the CES-D instrument is a well validated measure of depressive symptomatology [22,23]. The prevalence of MD in community samples of older adults ranges from 1-5%, with the majority of studies reporting prevalence in the lower end of the range [38-41]. It is therefore likely that the CES-D overestimated the prevalence of MD and our measure of depression represents both major and subthreshold depression. Another limitation is that our primary conclusions were drawn from the SOH group which only included 20 individuals; the potential for spurious findings therefore exists. It must also be acknowledged that in some participant’s vestibular dizziness rather than hypotension could have caused the symptoms of dizziness although in our study all participants who reported symptoms had a clinically significant drop in blood pressure. Moreover, dizziness may also be a feature of anxiety which is highly co-morbid with depression in this age group [42].

## Conclusion

In summary, we have identified an association between symptomatic OH and depression in a large community-based sample of older adults. Symptomatic orthostatic hypotension may be an important, but not widely recognised, risk factor for late-life depression which needs to be considered in studies examining the relationship between vascular disease and depression. Clinically, our results are important as they show that the relationship between SOH and depression is not limited to clinical samples but extends to the general population and potentially individuals with subthreshold depression. Clearly longitudinal studies are required to clarify the direction of this association.

## Competing interests

None of the authors have any financial or non-financial competing interests to disclose.

## Authors’ contributions

All authors were involved in the conception and design, analysis and interpretation of data. COR drafted the article and PK HC BL and RAK revised and edited the manuscript. GS provided statistical input. All authors gave final approval of the version to be published.

## Pre-publication history

The pre-publication history for this paper can be accessed here:

http://www.biomedcentral.com/1471-244X/13/266/prepub
